# Catamenial pneumothorax in Ghana: case report and literature review

**DOI:** 10.11604/pamj.2019.33.287.14187

**Published:** 2019-08-06

**Authors:** Isaac Okyere, Paul Sedem Komla Glover, Paa Kobina Forson, Perditer Okyere, Delali Blood-Dzraku

**Affiliations:** 1 Department of Surgery, School of Medicine and Dentistry, College of Health Sciences, Kwame Nkrumah University of Science and Technology and Komfo Anokye Teaching Hospital, Kumasi, Ghana; 2 Department of Emergency Medicine, Komfo Anokye Teaching Hospital, Kumasi, Ghana; 3 Department of Internal Medicine, School of Medicine and Dentistry, College of Health Sciences, Kwame Nkrumah University of Science and Technology and Komfo Anokye Teaching Hospital, Kumasi, Ghana; 4 MedFocus International, N0.39 Sam Nujoma Road, North Ridge, Accra, Ghana

**Keywords:** Catamenial, oral contraceptives, pneumothorax, haemoperitoneum, pleurodesis, catamenial pneumothorax

## Abstract

Catamenial pneumothorax is a rare condition that is often misdiagnosed. It is defined as spontaneous pneumothorax occurring within 72 hours before or after onset of menstruation. Etiology is unknown but could be linked to endometriosis. Pleural ablation via thoracoscopy and hormonal therapy are mainstay treatment options to avoid recurrence. We present a case of a young adult female who experienced gradual painless abdominal distention that resolved spontaneously after each menses twelve years post menarche. She was first seen at a peripheral facility where laparotomy undertaken was negative for suspected ectopic pregnancy. However, a bleeding omental mass was noticed and a biopsy taken. Histopathology reported it as an endometriotic tissue. The patient subsequently had recurrent cyclical chest pains and breathlessness leading to the diagnosis of catamenial pneumothorax. She had chemical pleurodesis done with sterile talc after chest tube drainage and has been well over two years now.

## Introduction

Spontaneous pneumothorax associated with menstruation is called catamenial pneumothorax. It is defined as spontaneous pneumothorax occurring within 72 hours before or after onset of menstruation. It is a rare condition that is often misdiagnosed and usually associated with endometriosis. We report a case of a young woman with recurrent catamenial pneumothorax treated with chest tube drainage, sterile talc pleurodesis and hormonal therapy. Recurrent nature of the condition causes significant morbidity. Correct diagnosis will lead to effective treatment and prevention of recurrence.

## Patient and observation

**Figure 1:**
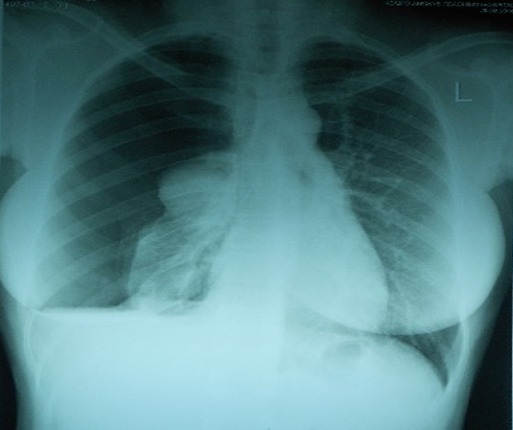
Chest x-ray showing massive right pneumothorax with right lung collapse and minimal effusion

**Figure 2:**
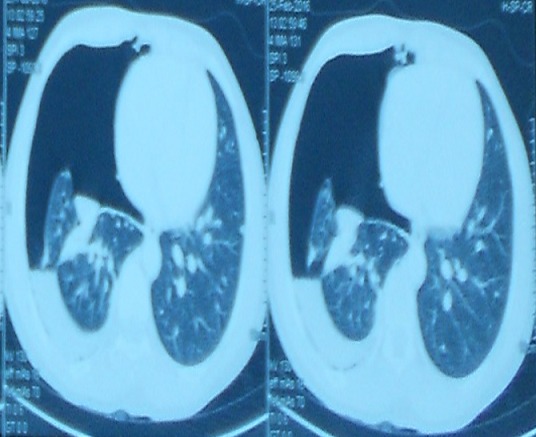
The CAT scan showing massive right pneumothorax with right lung collapse and minimal effusion

**Figure 3:**
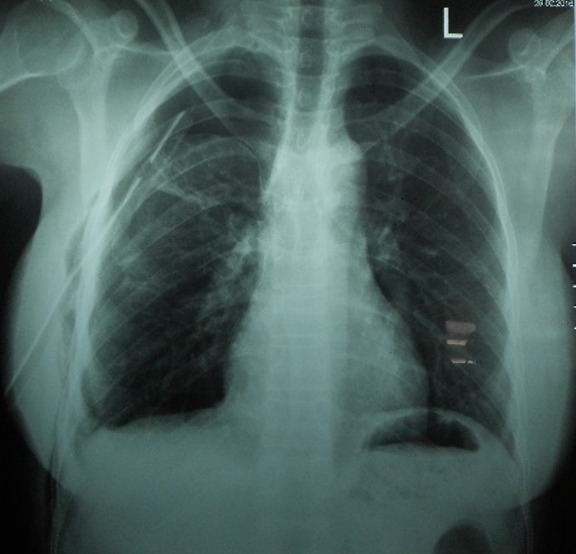
A repeat chest x-ray after 48 hours showing adequate re-expansion

A 25 year old nursing student presented to the Emergency Department with right sided pleuritic chest pain and exertional dyspnoea. Patient had menarche at 12 years and has been well until she started experiencing cyclical painless abdominal distension when she was 21 years. This was not associated with any gastrointestinal symptoms and therefore was initially managed as dysmenorrhea. In April 2011, she became dizzy, severely lethargic and disoriented during her menses. She was initially seen at a peripheral hospital where she had a laparotomy after an ultrasound done confirmed a haemoperitoneum though a urine pregnosticon test was negative. At laparotomy the ovaries, fallopian tubes and uterus were found to be normal but a bleeding mass was noticed on the omentum which was excised for histopathology which reported an endometriotic tissue. Subsequently, patient was well and had no repeated symptoms until March 2015 when she experienced heaviness in the right chest with easy fatigability and dyspnoea during her menses. A right haemothorax was diagnosed and a chest tube inserted draining 2 litres of coffee-ground coloured fluid over 48 hours at the same peripheral hospital. She was subsequently referred to do a Chest CT Scan for specialist care after being started on oral contraceptive pills (OCPs). Her past medical and surgical history was only significant for an epigastric hernia repair in 2007. Patient was not sexually active at the time she first had symptoms. Her usual menstrual cycle was 28 days with a moderate bleed lasting 5-6 days without dysmenorrhea. The menstrual cycle got changed in 2011 to 3 days of heavy bleed with clots and dysmenorhoea. The cycle became normal when she was started on OCPs. She had no family history of any bleeding disorder or endometriosis. Her vitals on presentation were stable including SpO2 of 94% on room air however there was decreased chest expansion and absent breath sounds over the right hemithorax. The CAT scan and chest x-ray showed massive right pneumothorax with right lung collapse as shown in Figure 1 and Figure 2. Subsequently, tube thoracostomy was done using a size 28 FG chest tube which was inserted in the 4thintercostal space midpoint between the anterior axillary line and the mid-axillary line draining gush of air. Chemical pleurodesis with 4 grams of sterile talc diluted to 50mls with normal saline was administered into the pleural space via the chest tube using a 60cc bladder syringe. The chest tube was removed after 48 hours following full lung expansion confirmed clinically and radiologically as shown Figure 3. She was then discharged for gynaecology follow up. She is doing well without recurrence for over two years.

## Discussion

Catamenial pneumothorax is recurrent accumulation of air in the pleural cavity in women of reproductive age without concomitant respiratory diseases. The sine qua non criterion is the occurrence of the pneumothorax in the period of 72 hours before or after the menses. Additional criteria include characteristic pleural lesions, right-sided occurrence and coexistence of endometriosis [1]. The features in the above definition were consistent with the findings in the case. It has always been considered a rare cause of primary spontaneous pneumothorax with a reported incidence of 2.8-5.6% [2]. This is probably the first reported case from Kumasi, Ghana as far as we know. Tettey *et al*. in [3] report the first of such cases in Accra in 2005 with a subsequent review of twelve cases by same Tettey *et al.* in 2013 in Ghana [3]. Recent studies suggest that this incidence in reality is around 30% [2], probably due to increased awareness. According to Marjanski T and his team [1], the etiopathogenesis of catamenial pneumothorax is explained by the following theories: physiological, migrational, microembolic-metastatic and the diaphragmatic theory of air “passage” as shown in Table 1, but none of them can fully explain the different manifestations of thoracic endometriosis syndrome (TES) which include catamenial pneumothorax, catamenial haemothorax, catamenial haemoptysis, pulmonary nodule, catamenial pneumomediastinum and isolated chest pain [4].

**Table 1:** Theories for the development of thoracic foci of endometriosis

Physiological	High concentration of prostaglandin F2 during menses may cause blood vessels and bronchioles to contract, which leads to alveolar rupture and development of pneumothorax.
Migrational	Endometrial cells may migrate from the uterus, through the fallopian tubes, into the lesser pelvis and further, into the area of the diaphragm. The cyclical proliferation and necrosis of endometrial cells may injure the diaphragm, enabling the cells to migrate further into the chest and the visceral pleura, which may lead to pulmonary alveolar injury and pneumothorax.
Microembolic-metastatic Diaphragmatic	Metastatic spread or pulmonary microembolization from endometrial cells through blood or lymph vessels. Necrosis of subpleural interstitial endometriotic foci leads to pneumothorax; foci located more centrally cause hemoptysis.
Diaphragmatic theory of air “passage”	Air passes through the uterus and fallopian tubes into the peritoneal cavity and, through diaphragmatic fenestrations, into the pleural cavity.

Endometriosis seems to play the most important role in the development of catamenial pneumothorax. Catamenial pneumothorax is found to be associated with 31-50% of pelvic endometriosis [1] as was found in this patient. The pneumothorax can progress to tension pneumothorax which is potentially fatal. Treatment options for catamenial pneumothorax include: aspiration where air is removed manually by way of a catheter or needle (catheter or needle thoracocentesis). A similar approach is the use of a one-way Heimlich valve. The second option is tube thoracostomy which involves the passage of a chest tube/chest drain into the pleural space with application of suction and water seal drainage until the lung re-expands. The third option is chemical sclerosis or pleurodesis where a chemical agent or sclerosant is inserted through the chest tube after chest drainage to cause chronic inflammation and scarring of the pleural surfaces promoting adhesions to form between the lung and chest wall, thereby discouraging future collapses. Chemical pleurodesis used alone to treat catamenial pneumothorax, without other surgical repairs and/or hormonal therapy has been found to have a significant failure rate [5]. The fourth option is endometrial suppression hormonal therapy which involves the use of progestins and gonadotropin releasing hormone agonists (GnRH) most commonly, to induce chemical menopause [6, 7]. This is done after chest drainage. Our patient underwent chest tube drainage and chemical pleurodesis as the mode of management. The sixth option is the use of thoracoscopy/Video-assisted thoracic surgery (VATS) which utilizes a fiber optic scope to visualize the lung, pleura and diaphragm. Diaphragm repair, bleb resection and pleurodesis can all be done during this procedure [2]. Then pleural abrasion which is mechanical pleurodesis involves abrading or “scrubbing” of the pleural surfaces during thoracoscopy or thoracotomy, so that resulting inflammation will form adhesions between the lung and pleural surfaces. Literature shows that pleurodesis used as a singular treatment, without other surgical repair or hormonal therapy, has a high failure rate [8]. Our patient has been free from recurrence after two years, having had the chest tube insertion and pleurodesis.

Another surgical option is thoracotomy with pleurectomy. Thoracotomy is performed to view the lung, diaphragm and pleura directly by opening the lung cavity. A pleurectomy is the removal of the pleura, which is designed to encourage adhesion of the lung directly to the chest wall. During thoracotomy, diaphragmatic perforations or fenestrations repair using polymesh can also be done [7]. This procedure was introduced in 2003 and involves a vicryl-type mesh which is placed over the entire diaphragm, to cover any small fenestrations that may not be seen by the surgeon [7]. The Vicryl material allows for tissue in-growth forming substantial scar tissue over the diaphragm. Recent articles indicate that this procedure is being used in Europe and US, and has been found to be especially successful when used in conjunction with hormonal therapy [8]. The other minimally invasive option is dual laparoscopic diaphragm evaluation and repair. Understanding the complex nature of this condition, thoracic surgeons and gynecologists utilize a dual laparoscopic procedure, whereby both the anterior and posterior of the diaphragm are evaluated for endometrial progression. This tandem approach reportedly improves chances of successful repair, when lesions are removed from both sides. Lastly a bilateral salpingo-oophorectomy through laparotomy or laparoscopy to remove both ovaries to induce surgical menopause, thereby limiting estrogen production and suppressing endometrial implants from bleeding [1] is also effective. Cessation of the menstrual cycle has shown to be an effective treatment for catamenial pneumothorax, as long as immediate estrogen replacement is withheld [9]. So for the management of catamenial pneumothorax, a chest tube should be inserted after clinical assessment. This is followed with chemical pleurodesis and continued with hormonal therapy. This combinational therapy is relatively effective in less resource centres.

## Conclusion

Catamenial pneumothorax should be suspected in every young woman presenting with pneumothorax or haemopneumothorax during menses. Diagnosis should be confirmed. Chest tube drainage releases the air and allows lung re-expansion. Chemical pleurodesis combined with hormonal therapy for at least six months may be adequate and effective in less resource centres. Surgery combined with hormonal therapy is the best option.

## Competing interests

The authors declare no competing interests.
